# The link of adverse childhood experiences and attachment to maladaptive personality traits in adults diagnosed with substance use disorder

**DOI:** 10.1186/s40359-025-03331-6

**Published:** 2025-08-27

**Authors:** Eva Migalova, Jana Furstova, Jozef Hasto, Peter Tavel, Natalia Kascakova

**Affiliations:** 1 Olomouc University Social Health Institute (OUSHI), Palacky University in Olomouc, Univerzitní 22, Olomouc, 771 11 Czech Republic; 2 Psychiatric-Psychotherapeutic Outpatient Clinic, Pro mente sana, Heydukova 27, Bratislava, 811 08 Slovakia

**Keywords:** Substance use disorder, Personality, Attachment, Adverse childhood experiences

## Abstract

**Background:**

Maladaptive personality traits are often associated with adverse childhood experiences and substance use disorders. At the same time, there is a multidirectional relationship between experiencing life stressors and maladaptive personality traits. A variable that also enters these relationships is relational attachment, which has an impact on an individual’s overall functioning. We aimed to examine these interconnections in a clinical sample.

**Methods:**

A clinical sample of adult patients with substance use disorder (*n =* 63, m_age_ = 38.87 ± 10.34 years, 71.43% men) was assembled. The data on adverse childhood experience (Adverse Childhood Experience International Questionnaire; ACE-IQ), attachment anxiety and avoidance (Experiences in Close Relationships – Revised; ECR-R), maladaptive personality traits (Personality Inventory for DSM-5 Short Form; PID-5-SF) were collected, and associations between variables were assessed by correlations and exploratory network analysis.

**Results:**

Adverse childhood experiences were reported by 92% of patients in the clinical sample. Spearman correlation analysis revealed significant associations between attachment anxiety and the PID-5 domains of Negative Affectivity, Antagonism, and Disinhibition. Attachment avoidance correlated with Detachment and Disinhibition. Specific ACEs, such as emotional neglect, emotional abuse, and parental loss, were significantly associated with Psychoticism and other maladaptive personality traits. Exploratory network analysis visualized robust links between attachment dimensions and personality traits, with Negative Affectivity, Detachment, and Disinhibition emerging as central nodes. These findings suggest that maladaptive personality traits serve as a key connecting point between early adversity and relational patterns in individuals with substance use disorders.

**Conclusions:**

The findings underscore the clinical relevance of maladaptive personality domains in understanding how adverse childhood experiences shape adult relational patterns in individuals with substance use disorders. Targeting these personality domains in assessment and intervention may enhance the effectiveness of therapeutic approaches addressing both addiction and attachment-related difficulties. Integrating this perspective into treatment planning could support more individualized interventions, particularly for patients with histories of early adversity. Furthermore, findings point to the potential benefit of trauma-informed and personality-focused therapeutic approaches to address underlying relational and emotional regulation difficulties. Sufficient preventive programs for maltreated children and youth should be a topic in question.

## Introduction

Adverse childhood experiences (ACEs) considerably increase the likelihood of mental illness, health risk behaviors, and chronic physical illnesses. Given their pervasive and enduring impact, ACEs represent a serious threat to long-term health and psychosocial functioning [1–3]. A comprehensive meta-analysis [4] involving over 250,000 participants showed that individuals who had experienced four or more ACEs had markedly increased odds of adverse health outcomes compared to those without such experiences. These included moderate to strong associations with mental illnesses, tobacco use, and problematic alcohol consumption. Similarly, the original ACE study [3] demonstrated that individuals exposed to multiple adverse experiences had a four- to twelve-fold increased risk for alcoholism, drug abuse, depression, and suicide attempts. These findings have since been replicated in various populations. A representative study in Slovakia [5] confirmed that childhood maltreatment—specifically emotional, physical, and sexual abuse—strongly predicted alcohol use disorder in adulthood. Even stronger associations were observed for problematic drug use [6, 7] and both interpersonal and self-directed aggression [8]. In line with international data, another research [9] also supported the observation that ACEs are more prevalent among individuals with substance use disorders (SUD) than in the general population.

A factor positively associated with negative health behavior is maladaptive personality traits, possibly rooted in childhood maltreatment [10]. In a nationwide study [11], the accumulation of various forms of maltreatment raised the likelihood of developing antisocial, avoidant, borderline, narcissistic, paranoid, schizoid, and schizotypal personality disorders. Other studies confirmed that childhood adversities most consistently predicted personality disorders from clusters A and B—most notably schizotypal, antisocial, borderline, and narcissistic profiles [12]. In recent years, there has been a growing emphasis on understanding personality pathology using dimensional models, particularly the Alternative Model for Personality Disorders (AMPD) introduced in DSM-5 Section III [13]. This model conceptualizes maladaptive personality traits along five broad domains—Negative Affectivity, Detachment, Antagonism, Disinhibition, and Psychoticism. Unlike traditional categorical diagnoses, which define PDs based on the presence of specific criteria sets, the AMPD focuses on assessing traits dimensionally. It also shows notable convergence with Section II PDs, as high scores in AMPD domains often align with prototypical profiles of Cluster A, B, and C PDs. Thus, even when not diagnosing PD per se, AMPD provides clinically meaningful insights into the pathological configurations of personality functioning.

The current study applies this dimensional trait-based framework to a clinical sample of individuals with SUD, aiming to explore how specific maladaptive personality traits may be linked to ACEs and attachment type. While the AMPD includes both trait domains (Criterion B) and severity of dysfunction (Criterion A), our focus lies on the former. Existing literature suggests that maladaptive personality traits are significantly associated with the development and maintenance of SUD [14]. For example, disinhibition has been linked to impulsive substance use [15], while negative affectivity may contribute to emotion-driven consumption [16]. By situating these traits within the context of early adversity and attachment style, we aim to better understand how developmental risk factors translate into enduring vulnerabilities relevant for addiction treatment and prevention.

Attachment style reflects an individual’s internalized expectations about self and others in close relationships, shaped predominantly by early caregiving experiences. As such, it functions as a developmental bridge between early adversity and adult relational and emotional functioning [17]. Attachment dimensions—anxiety and avoidance—are not static traits but regulatory strategies that influence how individuals cope with distress, manage emotions, and seek or avoid interpersonal support [18]. Insecure attachment, which often emerges as a developmental consequence of adverse caregiving environments, represents a key mechanism linking early adversity to later psychopathology [19, 20]. Beyond its origins, insecure attachment has been robustly associated with impairments in affect regulation, heightened interpersonal reactivity, and poor stress tolerance [21] — all of which are central vulnerabilities in both maladaptive personality functioning and SUD. For instance, a meta-analysis [22] found a positive link between attachment security and children’s top-down self-regulation capacities, reinforcing its role in shaping core emotional and behavioral competencies. These patterns can persist into adulthood and shape how individuals cope with distress, form relationships, and regulate internal states [23]. Although attachment style can evolve through significant relational experiences, longitudinal research indicates that it retains moderate stability across the lifespan [24, 25]. Attachment is thus not only a product of early developmental contexts but also a relatively enduring individual difference variable. Moreover, from a clinical standpoint, understanding a patient’s attachment style can guide how clinicians build therapeutic rapport, interpret transference patterns, and tailor interventions to meet underlying relational needs. Ignoring attachment would mean disregarding a major component of how early relational trauma gets expressed and maintained in adulthood.

Building on prior research [26] that has examined the links between ACEs, attachment insecurity, and personality pathology, the present study explores how these domains interact within a shared dimensional framework. Although the study is exploratory in nature, our hypotheses are guided by both existing literature and the authors’ clinical experience with individuals diagnosed with SUD, who often present with complex profiles involving early relational trauma, insecure attachment, and maladaptive personality traits. We anticipate that specific types of ACEs would be associated with specific maladaptive trait domains and attachment anxiety and avoidance, would be meaningfully related to these personality dimensions. While these associations have been explored in general or mixed psychiatric populations, few studies have investigated them in clinical SUD samples using a dimensional, trait-based model. Unlike studies focused on categorical personality disorder diagnoses, our research emphasizes personality traits that reflect enduring patterns of emotional, interpersonal, and cognitive dysfunction—even when these traits fall below diagnostic thresholds.

## Methods

### Research sample and data collection

Clinical sample data were collected in mental health facilities specialized in substance abuse treatment in Slovakia between October 2021 and April 2022. Computer-assisted personal interviewing [27] was used for data collection, and data were obtained by trained psychologists and psychiatrists employed at the departments. The study protocol was reviewed and approved by the Scientific Ethics Committee of Palacky University Olomouc (no. 2019/05) and the P. Pinel Psychiatric Hospital Ethics Committee (no. PAP-20), ensuring compliance with established ethical guidelines, including those outlined in the Declaration of Helsinki. Prior to data collection, each participant provided written informed consent, and confidentiality measures were strictly upheld. All subjects were informed about the purpose of the study and the possibility to terminate their participation at any time. The original research population consisted of 74 hospitalized patients. The primary diagnosis was determined by the attending psychiatrist at admission, based on DSM-5 criteria and clinical judgment, with priority given to the disorder most relevant to the treatment focus. Those patients whose primary diagnosis was gambling disorder were excluded (*n* = 11). Our final research population consisted of 63 patients. Of this number, 27 patients had a primary psychiatric diagnosis of an alcohol use disorder, and 36 patients were diagnosed with a psychoactive substance use disorder other than alcohol (opioids, amphetamines, polysubstance drug use). The dataset supporting the conclusions of this article is freely available together with materials of the study at this link in OSF repository: https://osf.io/5g8p4/?view_only=06222581a5b44559b61d5d885677185e.

## Measures

### Sociodemographic characteristic

The patients’ sociodemographic data were collected, including sex (male, female), age, education (primary, skilled operative, high school, university), and marital status (single, married, divorced, widow/widower, unmarried partner).

### Maladaptive personality traits

The degree of maladaptive personality traits was measured using the PID-5 [28] in its shortened 100-item form [29]. Shortened Slovak version was created by extracting particular items from linguistically validated translation of the inventory [30]. This self-assessment inventory is made up of 100 statements that the patient rates on a scale from 0 to 3, according to how truthful they are about them. It contains 25 aspects of personality, which in various combinations make up the 5 final personality domains (negative affect, disinhibition, antagonism, detachment, and psychoticism). The inventory retains good psychometric properties even in its short form [31, 32] and in test-retest [33]. The reliability was calculated using Cronbach’s alpha. Cronbach’s alpha was 0.864 for negative affect, 0.845 for detachment, 0.842 for antagonism, 0.830 for disinhibition, and 0.823 for psychoticism. These values indicate that the PID-5-SF domains are reliably measured and suitable for capturing maladaptive personality traits in individuals with substance use disorders.

### Adverse childhood experiences

Adverse childhood experiences were mapped retrospectively using the ACE-IQ consisting of 13 key areas [34]. They include emotional neglect and abuse, physical neglect and abuse, sexual abuse, alcoholism in a family, violence in a family, a family member in prison, a family member diagnosed with depression or other mental disorders, and the disappearance of a parent through divorce, death, or abandonment, bullying, collective (war, terrorism, political or ethnic conflict, genocide, coercion, disappearances, torture, and organized criminal violence), and community violence (in residency). The items can be either scored as binary where the respondent answers with yes or no in the means of the presence of ACEs, or frequency, where the answer provides information about possibly repeated occurrence of the ACEs. The ACE composite score was calculated according to WHO guidelines [35] using the frequency-based scoring method. Participant received 1 point for every reoccurring ACE, yielding a score from 0 to 13.

### Attachment

The statements in this 14-item Slovak version of the Experiences in Close Relationships Revised Questionnaire ECR-R [36] describe how the individual feels about close relationships. For each statement (e.g., “My desire for a very close relationship sometimes scares people away.” or “I usually talk about my problems and worries with my spouse/partner.“) the respondent is asked to express agreement or disagreement by selecting a number on a scale of 1 to 7. The results point at the levels of attachment anxiety and avoidance perceived by the individual. The scale used in this study demonstrated satisfactory internal consistency. Cronbach’s alpha was 0.816 for the attachment anxiety subscale and 0.758 for the attachment avoidance subscale, indicating acceptable to good reliability of both dimensions.

### Statistical analyses

Statistical analyses were performed using the statistical software package JASP version 0.16.2 (JASP Team, University of Amsterdam, The Netherlands) and R statistical software [37]. Characteristics of the research sample were quantified using descriptive statistics. A comprehensive correlation matrix was constructed to examine the associations among ACEs, attachment anxiety and avoidance, and the maladaptive personality domains. Network Analysis [38] was applied as an exploratory tool to examine the underlying structure of the data. This approach is grounded in graph theory, where variables are depicted as nodes and the relationships between them as edges, representing conditional associations while accounting for all other variables in the dataset. In these undirected networks – also referred to as pairwise Markov random fields – nodes that are not connected can be interpreted as conditionally independent. To estimate the network, we used the qgraph package in R [39] along with the EBICglasso estimator [40], which applies regularization techniques to reduce the inclusion of potentially spurious connections. Node centrality metrics were computed to evaluate the relative importance of individual variables within the network.

## Results

### Background characteristics

The background characteristics of the clinical sample are presented in Table 1. There were 27 AUD patients, 63% males, with a mean age of 42.2 years (SD = 11.06). The OSUD group comprised 36 patients, 77,78% males, with a mean age of 35.6 years (SD = 8.55).

**Table 1:** Sociodemographic characteristics of the SUD sample

Characteristic	*n* (%)
**SUD sample**	63 (100)
**Sex**	
Male	45 (71.43)
Female	18 (28.57)
**Age**	
18–34 years	23 (36.51)
35–54 years	34 (53.97)
55–68 years	6 (9.52)
**Education**	
Primary	15 (23.81)
Skilled operative	17 (26.98)
High school	23 (36.51)
College	8 (12.70)
**Marital status**	
Single	31 (49.20)
Married	12 (19.05)
Divorced	7 (11.11)
Widow/Widower	5 (7.94)
Unmarried partner	8 (12.70)

Note: n = number of participants

### Occurrence of adverse childhood experiences

Overall, 58 patients (92%) in our research sample experienced at least one reoccurring adverse experience in childhood. Almost half of the patients (49%) experienced at least 1 to 3, and 43% experienced more than 4 ACEs. The average occurrence of ACEs in the sample was M = 3.37 (SD = 2.28). Table 2 shows the frequency of occurrence for individual ACEs in whole sample. The item “community violence” is not included in the figure since it has not been reported by any patient. The most frequent ACEs were “alcoholism in the family”, followed by “emotional neglect” and “loss of a parent” due to death, divorce, or any other form of physical separation from the child.

**Table 2:** Frequency of reoccurring adverse childhood experiences of the SUD sample

Adverse childhood experience	*n* (%)	
Alcoholism in family	36 (57.14)	
Emotional neglect	34 (53.97)	
Loss of parent	30 (47.62)	
Bullying	29 (46.03)	
Family violence	27 (42.86)	
Emotional abuse	19 (30.16)	
Collective violence	18 (28.57)	
Physical abuse	13 (20.64)	
Mental illness in family	10 (15.87)	
Physical neglect	10 (15.87)	
Imprisonment in family	9 (14.29)	
Sexual abuse	4 (6.35)	

Note: n = number of participants

### Correlations analysis

**Fig. 1:**
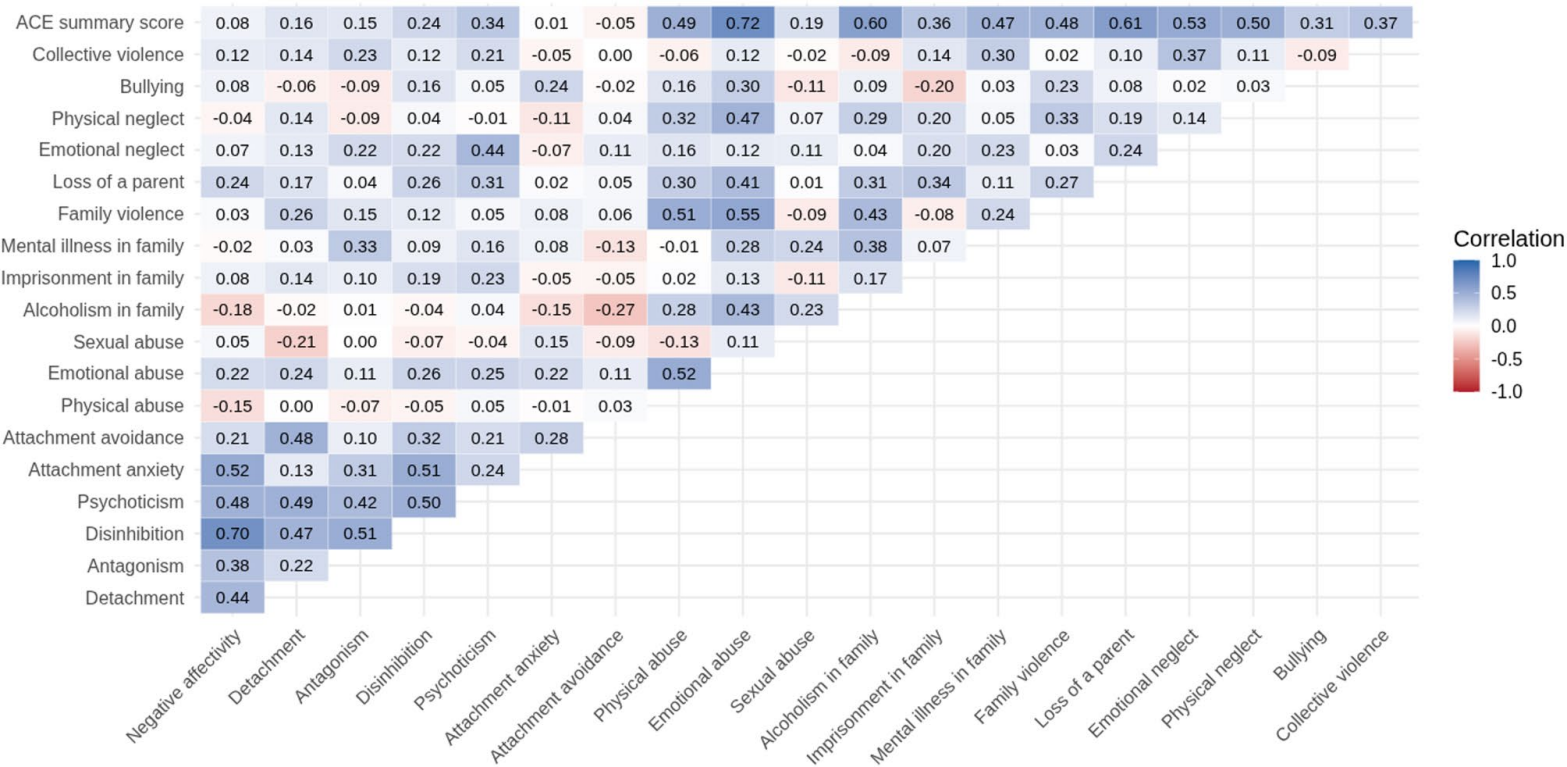
Spearman correlation matrix (ρ)

Spearman correlation analysis revealed several statistically significant relationships between maladaptive personality traits, attachment styles, and ACEs. Attachment anxiety showed significant positive correlations with several PID-5 domains: Negative Affectivity (ρ = 0.52, *p* < 0.001), Antagonism (ρ = 0.31, *p* = 0.013), and Disinhibition (ρ = 0.51, *p* < 0.001). Attachment avoidance was significantly associated with Detachment (ρ = 0.48, *p* < 0.001) and with Disinhibition (ρ = 0.32, *p* = 0.012). Regarding ACEs, significant positive correlations were found between: Detachment and Family violence (ρ = 0.26, *p* = 0.036), Antagonism and Mental illness in family (ρ = 0.33, *p* = 0.009), Disinhibition and Emotional neglect (ρ = 0.27, *p* = 0.036), and Loss of a parent (ρ = 0.26, *p* = 0.042). When it comes to Psychoticism, three of the ACEs were significantly associated: Emotional neglect (ρ = 0.44, *p* < 0.001), Emotional abuse (ρ = 0.25, *p* = 0.044), and Loss of a parent (ρ = 0.31, *p* = 0.014). Out of all five domains, Psychoticism correlated significantly with ACE summary score (ρ = 0.34, *p* = 0.006). A complete overview of all correlations is presented in Fig. 1.

### Network analysis

**Fig. 2:**
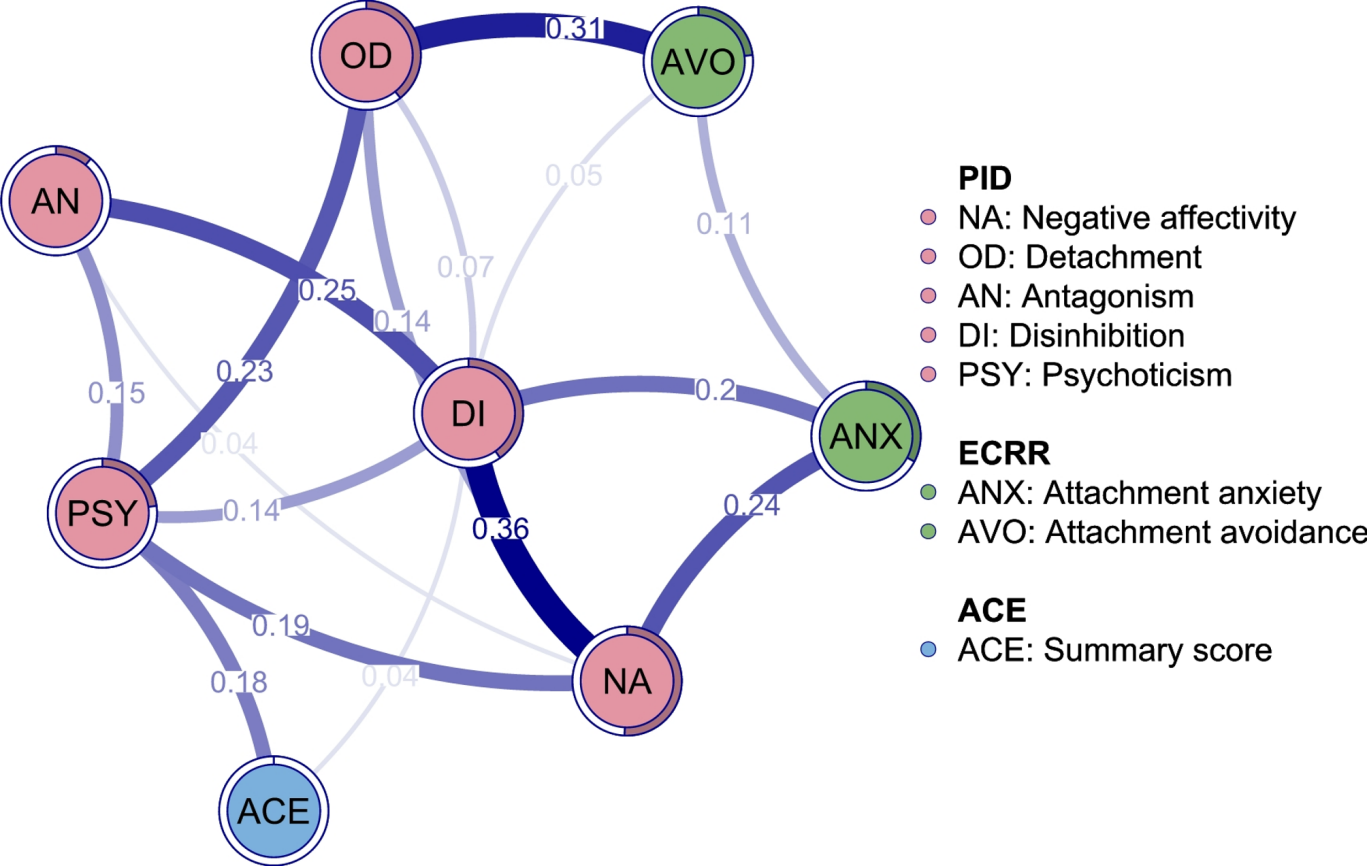
Network model of ACE summary score, attachment anxiety and avoidance (ECRR), and maladaptive personality domains (PID). Shaded areas (pies) surrounding nodes represent the predictability of the nodes

**Fig. 3:**
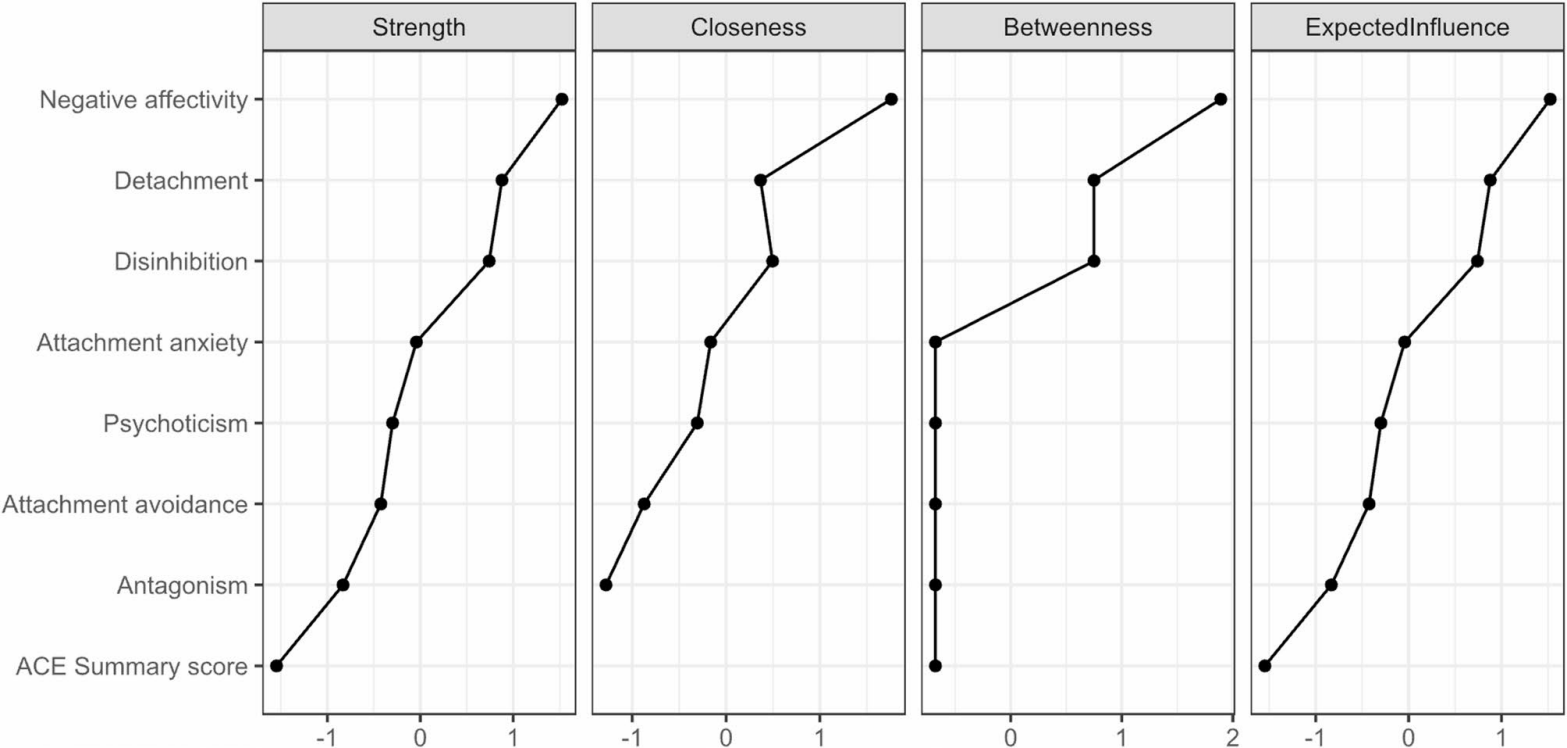
Node centrality measures of PID-5 domains, ACEs and attachment types. X-axes of centrality measures are scaled as Z-scores

To explore the associations between ACEs, attachment styles, and maladaptive personality traits in our sample, we performed an exploratory network analysis (Fig. 2). The resulting network contained only positive edges, indicating no negative conditional associations between variables. The PID-5 domains exhibited several interconnections. Attachment avoidance was linked to Detachment, while attachment anxiety showed associations with Negative Affectivity and Disinhibition. The strongest link of the ACE summary score was with Psychoticism. Among all variables, the PID-5 domains – particularly Detachment, Negative Affectivity, and Disinhibition – exhibited the highest centrality values (Fig. 3), indicating their prominent role within the network structure.

## Discussion

The present study aimed to explore the relationships between ACEs, attachment anxiety and avoidance, and maladaptive personality traits in patients with SUD in Slovakia. The findings offer insights into the potential configurations of psychological vulnerability present within this population. As anticipated, a high proportion of participants (92%) reported repeated exposure to at least one ACE, with 43% reporting four or more. When it comes to binary scoring, 98% of participants reported at least one ACE, 79% experienced four or more. These frequencies are consistent with previous studies documenting elevated rates of childhood adversity among individuals with SUD [3, 5, 9]. Importantly, recent data from a nationally representative Slovak population study [41] showed substantially lower rates of cumulative ACE exposure in the general population. Only 31% of participants reported four or more ACEs based on binary scoring, and just 7% based on frequency scoring. This striking difference underlines the relevance of investigating adversities in vulnerable clinical groups.

Our results indicate several statistically significant associations between specific ACE categories and maladaptive personality domains. For instance, Psychoticism was positively correlated with emotional neglect, emotional abuse, and parental loss. While these associations do not imply causality, their co-occurrence suggests that more severe personality disturbances may frequently emerge alongside relational trauma and early emotional deprivation. These findings are in line with previous work indicating that cumulative maltreatment increases the risk of Cluster A and B personality disorders [11, 12], and that specific forms of neglect and abuse contribute to the development of maladaptive personality traits [10].

Similarly, Antagonism, Disinhibition, and Detachment were each associated with distinct types of early adversity—such as family violence, mental illness in the family, and emotional neglect. This aligns with findings that ACEs are linked not only with SUD onset, but also with broader emotional and interpersonal dysregulation [4, 6].

Importantly, both attachment anxiety and avoidance showed significant correlations with specific personality domains. Attachment anxiety was positively related to Negative Affectivity and Disinhibition. Attachment avoidance, on the other hand, was related to Detachment, consistent with a defensive withdrawal from interpersonal closeness. These findings support theoretical models that view attachment as a relational regulation mechanism influencing affective and interpersonal functioning [18]. They also reflect the broader literature identifying attachment insecurity as a key factor in the developmental pathways from ACEs to adult psychopathology [17, 22, 24].

The network analysis provided an additional lens through which to examine the structure of these interrelationships. Among all nodes, Detachment, Negative Affectivity, and Disinhibition demonstrated the highest centrality scores—suggesting that they may occupy key positions within the psychological network observed in this sample. While these network patterns do not indicate causal sequencing, they help visualize the density and centrality of interconnections across variables.

Notably, although several bivariate correlations were found between specific variables, these associations were less prominent in the network analysis. This apparent discrepancy reflects key methodological differences between the two approaches. While correlation analysis captures general co-occurrence between variables, network analysis identifies only those associations that remain statistically meaningful when controlling for all other variables in the system. For example, bivariate correlations suggested a direct link between emotional abuse and disinhibition, the network analysis indicated that this association may be more plausibly explained through attachment mechanisms. Clinically, persons with a history of emotional abuse and neglect often develop an anxious (preoccupied) attachment style [42–45], characterized by heightened sensitivity to rejection, difficulties with affect regulation, and persistent fears about the availability of close others [21]. In adulthood, these vulnerabilities may manifest as impulsive behaviors, including substance use, which serve as maladaptive strategies to cope with overwhelming emotional states. Thus, what appears as a direct ACE–Disinhibition relationship in correlational analysis may, in fact, reflect a more complex developmental pathway in which anxious attachment mediates the expression of early adversity into disinhibited patterns of functioning.

Another illustration of this distinction can be seen in the case of Detachment. In the correlational analysis, Detachment showed a significant association with family violence, suggesting that experiences of violence in the household co-occur with elevated interpersonal withdrawal. In the network analysis, however, this direct association did not remain, and Detachment was instead primarily linked to avoidant attachment. Conceptually, this pattern may indicate that the impact of family violence on Detachment is not best captured as a direct pathway, but rather as one that operates through the development of avoidant relational strategies [46, 47]. In other words, while correlations highlight the co-occurrence of early adverse experiences and Detachment, the network model suggests that avoidant attachment may represent the more proximal mechanism through which such adversities are expressed in personality functioning.

The ACE summary score, as an aggregate of diverse adverse experiences, may influence psychological functioning indirectly—through overlapping variance with more central constructs such as attachment insecurity or maladaptive personality traits. These constructs may serve as mediating mechanisms or proximal expressions of early adversity, which could explain their higher centrality in the network.

### Strengths and limitations

While our study contributes to the understanding of the relationships between ACEs, attachment anxiety and avoidance, and maladaptive personality traits in patients with SUD in Slovakia, some limitations must be addressed. Due to the limited sample size in the AUD (*n* = 27) and OSUD (*n* = 36) subgroups, and in the interest of producing an interpretable network, we estimated a joint network model across the entire SUD sample. While we recognize potential differences between these groups, sample constraints did not allow for subgroup-specific networks. However, we acknowledge this as a limitation which also obstructs the generalizability of the study findings and encourage future research with larger samples to examine these distinctions in more detail. While the gender imbalance in our sample (predominantly male) reflects the epidemiological reality of clinical SUD populations, it may also limit the generalizability of our findings to female patients. Second, the study sample was limited to inpatients undergoing a residential rehabilitation addiction treatment program, which may not be representative of the general population with SUD. Third, since all questionnaires were self-reported, socially desirable answers could have been provided, distorting the results of the scales.

### Implications

From a clinical perspective, these findings underscore the importance of routinely assessing not only trauma history but also personality functioning and attachment patterns in individuals with SUDs. These factors are not passive background features but active mechanisms that shape treatment engagement, emotional regulation, interpersonal functioning, and vulnerability to relapse. Crucially, our capacity to differentiate subtypes of adversity—such as physical, emotional, and sexual abuse—enables us to make more precise assumptions about the long-term mental health impacts of childhood trauma. This granularity allows for the design and selection of targeted interventions that address specific developmental capabilities at risk, such as social behavior, emotional experiencing, self-regulation, and mentalizing. Failure to resolve these early experiences may significantly shape maladaptive trajectories into adulthood [48]. Thus, integrating trauma-informed and personality-focused models into addiction treatment is not only theoretically sound but also essential for tailoring interventions to the individualized profiles of patients with complex relational and developmental histories.

## Conclusion

Our findings highlight the high co-occurrence of adverse childhood experiences, insecure attachment dimensions, and maladaptive personality traits in a clinical population with substance use disorders. Although no causal inferences can be made, the observed associations suggest that specific configurations of early adversity, relational insecurity, and personality traits may cluster in clinically meaningful ways. These insights support the relevance of incorporating trauma-informed and personality-sensitive approaches to addiction care.

## Data Availability

The dataset supporting the conclusions of this article is freely available together with materials of the study at this link in OSF repository: https://osf.io/5g8p4/?view_only=06222581a5b44559b61d5d885677185e.

## Abbreviations

ACEs: Adverse childhood experiences
ACE–IQ: Adverse childhood experience international questionnaire
DSM–5: The diagnostic and statistical manual of mental disorders, fifth edition
ECR–R: Experiences in close relationships–revised
OSUD: Other substances use disorders
PDs: Personality disorders
PID–5–SF: Personality inventory for DSM–5 short form
PNPP: Psychiatric hospital of philipp pinel
SD: Standard deviation
SUD: Substance use disorder
